# Preoperative DLco and FEV_1_ are correlated with postoperative pulmonary complications in patients after esophagectomy

**DOI:** 10.1038/s41598-024-56593-2

**Published:** 2024-03-13

**Authors:** Taeyun Kim, Yeong Jeong Jeon, Hyun Lee, Tae Ho Kim, Seong Yong Park, Danbee Kang, Yun Soo Hong, Genehee Lee, Junghee Lee, Sumin Shin, Jong Ho Cho, Yong Soo Choi, Jhingook Kim, Juhee Cho, Jae Ill Zo, Young Mog Shim, Hong Kwan Kim, Hye Yun Park

**Affiliations:** 1 Division of Pulmonary and Critical Care Medicine, Department of Medicine, Kosin University Gospel Hospital, Kosin University College of Medicine, Busan, South Korea; 2 Department of Thoracic and Cardiovascular Surgery, Samsung Medical Center, Sungkyunkwan University School of Medicine, 81 Irwon-ro, Gangnam-gu, Seoul, 06351 Republic of Korea; 3 Department of Internal Medicine, Hanyang University College of Medicine, Seoul, South Korea; 4 Department of Thoracic and Cardiovascular Surgery, Chung-Ang University Hospital, Seoul, South Korea; 5 Center for Clinical Epidemiology, Samsung Medical Center, Sungkyunkwan University School of Medicine, Seoul, South Korea; 6 Department of Epidemiology and Medicine, and Welch Center for Prevention, Epidemiology, and Clinical Research, Johns Hopkins Bloomberg School of Public Health, Baltimore, MD USA; 7 Patient-Centered Outcomes Research Institute, Samsung Medical Center, Seoul, South Korea; 8 Department of Clinical Research Design and Evaluation, SAIHST, Sungkyunkwan University, Seoul, South Korea; 9 Division of Pulmonary and Critical Care Medicine, Department of Internal Medicine, School of Medicine, Samsung Medical Center, Sungkyunkwan University, 81 Irwon-ro, Gangnam-gu, Seoul, 06351 Republic of Korea

**Keywords:** Esophageal cancer, Esophagectomy, Pulmonary complications, FEV_1_, DLco, Cancer, Gastrointestinal diseases, Respiratory tract diseases

## Abstract

Limited information is available regarding the association between preoperative lung function and postoperative pulmonary complications (PPCs) in patients with esophageal cancer who undergo esophagectomy. This is a retrospective cohort study. Patients were classified into low and high lung function groups by the cutoff of the lowest fifth quintile of forced expiratory volume in 1 s (FEV_1_) %predicted (%pred) and diffusing capacity of the carbon monoxide (DLco) %pred. The PPCs compromised of atelectasis requiring bronchoscopic intervention, pneumonia, and acute lung injury/acute respiratory distress syndrome. Modified multivariable-adjusted Poisson regression model using robust error variances and inverse probability treatment weighting (IPTW) were used to assess the relative risk (RR) for the PPCs. A joint effect model considered FEV_1_%pred and DLco %pred together for the estimation of RR for the PPCs. Of 810 patients with esophageal cancer who underwent esophagectomy, 159 (19.6%) developed PPCs. The adjusted RR for PPCs in the low FEV_1_ group relative to high FEV_1_ group was 1.48 (95% confidence interval [CI] = 1.09–2.00) and 1.98 (95% CI = 1.46–2.68) in the low DLco group relative to the high DLco group. A joint effect model showed adjusted RR of PPCs was highest in patients with low DLco and low FEV_1_ followed by low DLco and high FEV_1_, high DLco and low FEV_1_, and high DLco and high FEV_1_ (Reference). Results were consistent with the IPTW. Reduced preoperative lung function (FEV_1_ and DLco) is associated with post-esophagectomy PPCs. The risk was further strengthened when both values decreased together.

## Introduction

Postoperative pulmonary complications (PPCs) occur in 16–67% of patients after esophagectomy, which accounts for two-thirds of the deaths associated with esophagectomy and affects the long-term survival rate in patients with esophageal cancer^1–7^. Therefore, to improve surgical treatment outcomes as well as the long-term survival rate, it is important to identify risk factors of PPCs in patients with esophageal cancer who are expected to undergo esophagectomy.

Lung function measurement is one of the important determinants for the risk stratification of patients who undergo thoracic surgery. Previous studies showed that forced expiratory volume in 1 s (FEV_1_) or diffusing capacity of carbon monoxide (DLco) could predict PPCs in lung cancer patients following lung resection surgery^8–10^. In addition, these measurements were found to be useful to identify a high-risk group in patients who undergo extra-pulmonary surgery^11,12^. However, in terms of PPCs after esophagectomy, studies mainly have focused on the types of surgery for predicting the occurrence of PPCs^5,13–15^. Although some studies have examined the relationship between lung function and post-esophagectomy PPCs^5,16–18^, these are limited by their study design (single center with a single surgeon), patient enrollments in the past, small numbers of participants, reliance on multiple imputation due to missing data, lack of consideration for individual components of PPCs. Moreover, it would be of value to consider lung function parameters together for the estimation of PPCs.

In this regard, this study aimed to evaluate the association between several preoperative lung function and the occurrence of PPCs and its components (atelectasis requiring bronchoscopic intervention, pneumonia, and acute lung injury [ALI]/acute respiratory distress syndrome [ARDS]) in patients with esophageal cancer who underwent esophagectomy.

## Results

### Patients’ characteristics

The baseline characteristics of 810 patients who underwent esophagectomy for esophageal cancer were summarized in Table 1. PPCs occurred in 19.6% (n = 159) of patients with esophageal cancer. Compared who did not develop PPCs, those who developed PPCs were more likely to be older, had more cardiovascular diseases and lower albumin, and underwent more thoracotomy. Preoperative lung function measurements, including FVC %pred, FEV_1_%pred, and DLco %pred were lower in patients who developed PPCs than those who did not develop PPCs.

**Table 1:** Baseline characteristics of patients with esophageal cancer who underwent esophagectomy by PPCs (N = 810).

	Patients who did not develop PPCs (n = 651)	Patients who developed PPCs (n = 159)	*P* value
Age, years	64 (58–70)	67 (62–73)	< 0.01
Sex, male	597 (91.7)	150 (94.3)	0.34
Body mass index, kg/m^2^	23.4 ± 2.9	23.2 ± 3.3	0.44
Smoking status			0.08
Never smoker	84 (12.9)	12 (7.5)	
Ever smoker	567 (87.1)	147 (92.5)	
Comorbidities			
Pulmonary comorbidities			
Asthma	12 (1.8)	4 (2.5)	0.53
Previous pulmonary tuberculosis	67 (10.3)	25 (15.7)	0.07
Interstitial lung disease	1 (0.2)	0 (0.0)	> 0.99
Extra-pulmonary comorbidities			
Diabetes mellitus	95 (14.6)	33 (20.8)	0.07
Hypertension	278 (42.7)	77 (48.4)	0.22
Cardiovascular disease	68 (10.4)	27 (17.0)	0.03
Laboratory findings			
Hemoglobin, g/dL	14.2 (13.3–15.0)	13.9 (12.9–15.0)	0.19
Albumin, g/dL	4.4 (4.2–4.6)	4.3 (4.1–4.5)	< 0.01
Creatinine, mg/dL	0.9 (0.8–1.0)	0.9 (0.8–1.0)	0.49
Pathologic stage			0.15
I	343 (52.7)	71 (44.7)	
II	177 (27.2)	47 (29.6)	
III	131 (20.1)	41 (25.8)	
Histologic type			0.39
Squamous cell carcinoma	618 (94.9)	155 (97.5)	
Adenocarcinoma	30 (4.6)	4 (2.5)	
Others	3 (0.5)	0 (0.0)	
Location of esophagus			0.42
Cervical/upper thoracic	81 (12.4)	26 (16.4)	
Middle thoracic	286 (43.9)	68 (42.8)	
Lower thoracic/esophagogastric junction	284 (43.6)	65 (40.9)	
Type of surgery			< 0.01
Open thoracotomy surgery	396 (60.8)	119 (74.8)	
VATS	88 (13.5)	12 (7.5)	
Robotic surgery	167 (25.7)	28 (17.6)	
Type of surgical approach			0.57
Transthoracic	633 (97.2)	153 (96.2)	
Trans-hiatal	15 (2.3)	5 (3.1)	
Others	3 (0.5)	1 (0.6)	
Lymph node dissection			0.06
Two-field or less	572 (87.9)	130 (81.8)	
Three-field	79 (12.1)	29 (18.2)	
Anastomosis site			0.10
Intrathoracic	349 (53.6)	97 (61.0)	
Cervical	298 (45.8)	60 (37.7)	
Abdominal	4 (0.6)	2 (1.3)	
Surgery time, hours	4.4 (3.8–5.0)	4.4 (3.8–5.3)	0.36
Preoperative pulmonary function test			
FVC, L	3.9 ± 0.7	3.6 ± 0.7	< 0.01
FVC, %predicted	93.0 ± 12.7	88.4 ± 13.2	< 0.01
FEV_1_, L	2.9 ± 0.6	2.5 ± 0.6	< 0.01
FEV_1_, %predicted	91.0 (82.0–101.0)	86.0 (76.0–96.5)	< 0.01
FEV_1_/FVC	0.7 (0.7–0.8)	0.7 (0.6–0.8)	0.01
DLco, %predicted	89.0 (77.0–100.0)	79.0 (68.5–92.0)	< 0.01

Data are presented as number (%) or mean (SD) or median (interquartile range).

PPCs, postoperative pulmonary complications; VATS, video-assisted thoracoscopic surgery; FVC, forced vital capacity; FEV_1_, forced expiratory volume in 1 s; DLco, diffusing capacity of the lung for carbon monoxide.

### The incidence of PPCs by FEV1%pred

**Figure 1:**
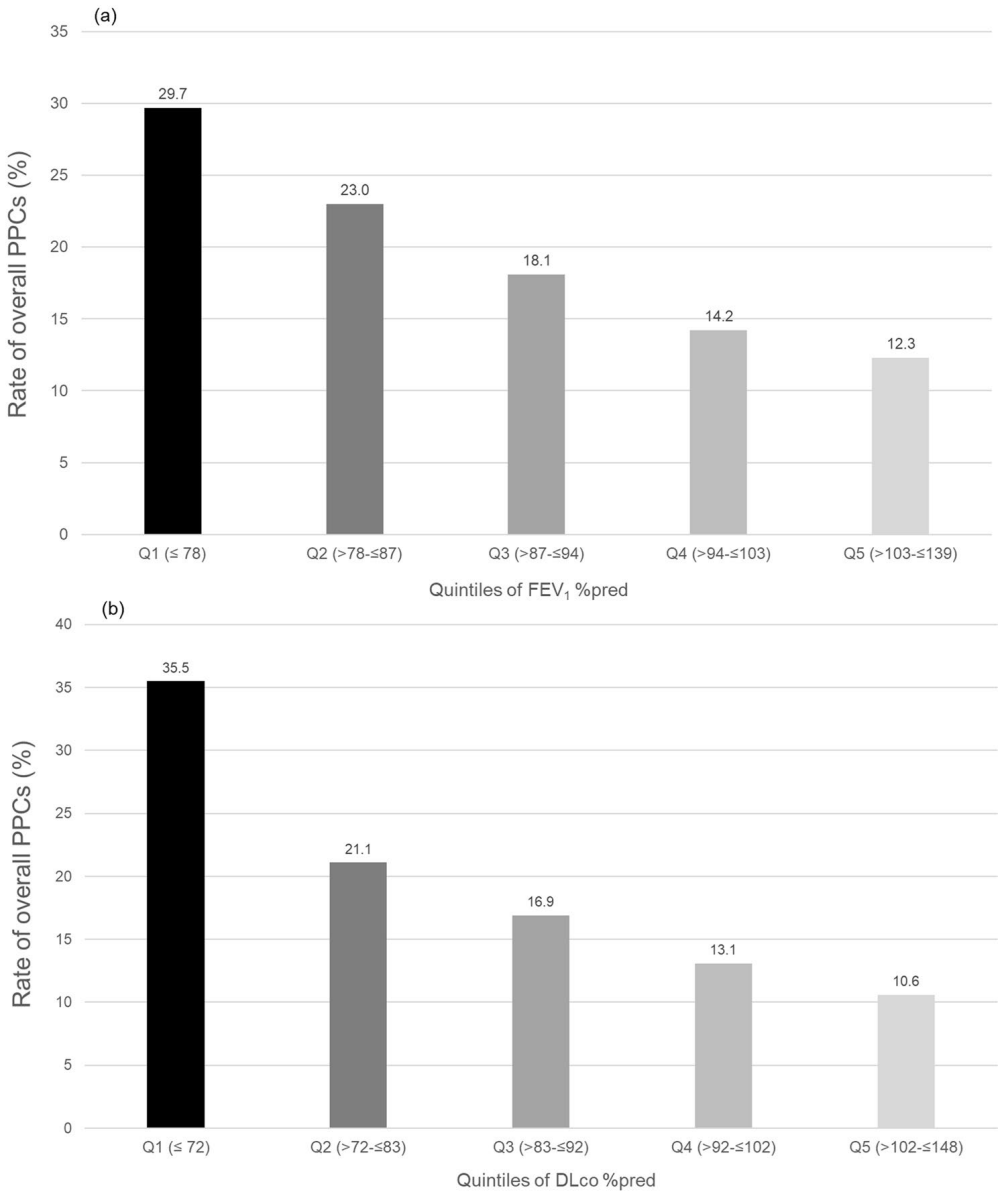
Post-esophagectomy pulmonary complications. (**a**) PPC by the quintiles of FEV_1_%pred, (**b**) PPC by the quintiles of DLco %pred. PPCs, postoperative pulmonary complications; FEV_1_, forced expiratory volume in 1 s; %pred, %predicted; DLco, diffusing capacity of the lung for carbon monoxide, Q1, the lowest quintile; Q5, the top quintile.

As shown in Fig. 1a and Table 2, the rate of overall PPCs tended to increase as FEV_1_%pred decreased (Q1 group, 29.7%; Q2 group, 23.0%; Q3 group, 18.1%; Q4 group, 14.2%; and Q5 group, 12.3%; *P* for trend < 0.01). The increasing trend in the incidence of pneumonia and ALI/ARDS was significant according to FEV_1_%pred.

**Table 2:** Incidence of PPCs by the quintiles of FEV_1_%pred and DLco %pred in patients with esophageal cancer who underwent esophagectomy.

	FEV_1_%pred						
	Total (%)	Quintile 1 (n = 165) ≤ 78	Quintile 2 (n = 165) > 78– ≤ 87	Quintile 3 (n = 171) 87 > – ≤ 94	Quintile 4 (n = 155) 94 > – ≤ 103	Quintile 5 (n = 154) 103 > – ≤ 139	*P* for trend
Overall PPCs	159 (19.6)	49 (29.7)	38 (23.0)	31 (18.1)	22 (14.2)	19 (12.3)	< 0.01
Atelectasis requiring bronchoscopic intervention	22 (2.7)	6 (3.6)	4 (2.4)	7 (4.1)	1 (0.6)	4 (2.6)	0.35
Pneumonia	132 (16.3)	43 (26.1)	28 (17.0)	24 (14.0)	20 (12.9)	17 (11.0)	< 0.01
ALI/ARDS	34 (4.2)	10 (6.1)	13 (7.9)	6 (3.5)	3 (1.9)	2 (1.3)	< 0.01

	DLco %pred						
	Total (%)	Quintile 1 (n = 166) ≤ 72	Quintile 2 (n = 171) > 72– ≤ 83	Quintile 3 (n = 160) > 83– ≤ 92	Quintile 4 (n = 153) > 92– ≤ 102	Quintile 5 (n = 160) > 102– ≤ 148	*P* for trend
Overall PPCs	159 (19.6)	59 (35.5)	36 (21.1)	27 (16.9)	20 (13.1)	17 (10.6)	< 0.01
Atelectasis requiring bronchoscopic intervention	22 (2.7)	9 (5.4)	3 (1.8)	4 (2.5)	3 (2.0)	3 (1.9)	0.09
Pneumonia	132 (16.3)	49 (29.5)	29 (17.0)	22 (13.8)	19 (12.4)	13 (8.1)	< 0.01
ALI/ARDS	34 (4.2)	14 (8.4)	11 (6.4)	6 (3.8)	1 (0.7)	2 (1.3)	< 0.01

Data are presented as number (%).

PPCs, postoperative pulmonary complications; FEV_1_, forced expiratory volume in 1 s; %pred, %predicted; DLco, diffusing capacity of the lung for carbon monoxide; Quantile 1, the lowest quintile; Quantile 5, the top quintile; ALI, acute lung injury; ARDS, acute respiratory distress syndrome; COPD, chronic obstructive pulmonary disease.

The relative risk (RR) of overall PPCs was highest in Q1 group of FEV_1_%pred followed by Q2, Q3, Q4, and Q5 (Table 3). This significance of trend remained after adjustment for covariables. Table 4 shows the RR of overall and individual PPCs in the low FEV_1_ group versus high FEV_1_ group. The RR of overall PPCs in the low FEV_1_ group was significantly higher than that of the high FEV_1_ group. For individual PPCs, the low FEV_1_ group had a significantly higher risk of pneumonia compared to the high FEV_1_ group. The results using the inverse probability treatment weighting (IPTW) were similar to the multivariable-adjusted model.

**Table 3:** The relative risk for PPCs in patients with esophageal cancer who underwent esophagectomy by the quintiles of FEV_1_%pred and DLco %pred.

	FEV_1_%pred					*P* for trend
	Quintile 1 (n = 165) ≤ 78	Quintile 2 (n = 165) > 78– ≤ 87	Quintile 3 (n = 171) > 87– ≤ 94	Quintile 4 (n = 155) > 94– ≤ 103	Quintile 5 (n = 154) > 103– ≤ 139	
Crude	2.41 (1.49–3.90)	1.87 (1.13–3.09)	1.47 (0.87–2.49)	1.15 (0.65–2.04)	Reference	< 0.01
Adjusted^a^	2.05 (1.24–3.38)	1.69 (1.02–2.82)	1.55 (0.92–2.62)	1.17 (0.66–2.06)	Reference	< 0.01
IPTW	2.72 (1.60–4.64)	1.98 (1.12–3.48)	1.66 (0.93–2.97)	1.30 (0.70–2.42)	Reference	< 0.01

	DLco %pred					*P* for trend
	Quintile 1 (n = 166) ≤ 72	Quintile 2 (n = 171) > 72– ≤ 83	Quintile 3 (n = 160) > 83– ≤ 92	Quintile 4 (n = 153) > 92– ≤ 102	Quintile 5 (n = 160) > 102– ≤ 148	
Crude	3.35 (2.04–5.48)	1.98 (1.16–3.38)	1.59 (0.90–2.80)	1.23 (0.67–2.26)	Reference	< 0.01
Adjusted^a^	2.98 (1.72–5.15)	1.88 (1.08–3.26)	1.59 (0.89–2.82)	1.24 (0.68–2.28)	Reference	< 0.01
IPTW	2.83 (1.63–4.92)	1.83 (1.01–3.30)	1.57 (0.84–2.93)	0.90 (0.45–1.79)	Reference	< 0.01

Data are presented as a ratio (95% confidence interval).

PPCs, postoperative pulmonary complications; FEV1, forced expiratory volume in 1 s; %pred, %predicted; Quintile 1, the lowest quintile; Quintile 5, the top quintile; DLco, diffusing capacity of the lung for carbon monoxide, IPTW, inverse probability treatment weight.

^a^Adjusted for age, sex, body mass index, smoking status (never and ever), chronic pulmonary disease, cardiovascular disease, albumin, pathologic stage (I, II, and III), tumor location (cervical/upper thoracic, middle thoracic, and lower thoracic/esophagogastric junction), type of surgery (open thoracotomy, video-assisted thoracoscopic, and robotic surgery), lymph node dissection (two-field or less and three-field), operation time.

**Table 4:** The relative risk for PPCs comparing the low pulmonary function group and the high lung function group using different cutoffs in patients with esophageal cancer who underwent esophagectomy.

	Model	FEV_1_%pred	
		Quintile 1 ≤ 78 (n = 165)	Quintile 2–5 > 78 (n = 645)
Overall PPC**s**	Crude	1.74 (1.30–2.33)	Reference
	Adjusted^a^	1.48 (1.09–2.01)	Reference
	IPTW	1.56 (1.13–2.16)	Reference
Atelectasis requiring bronchoscopic intervention	Crude	1.47 (0.58–3.69)	Reference
	Adjusted^a^	1.57 (0.55–4.48)	Reference
	IPTW	1.59 (0.58–4.40)	Reference
Pneumonia	Crude	2.04 (1.41–2.96)	Reference
	Adjusted^a^	1.76 (1.20–2.59)	Reference
	IPTW	1.77 (1.18–2.67)	Reference
ALI/ARDS	Crude	1.63 (0.79–3.34)	Reference
	Adjusted^a^	1.43 (0.65–3.11)	Reference
	IPTW	1.43 (0.65–3.17)	Reference

	Model	DLco % pred	
		Quintile 1 (n = 169) ≤ 72	Quintile 2–5 (n = 656) > 72
Overall PPCs	Crude	2.29 (1.74–3.01)	Reference
	Adjusted^a^	1.98 (1.46–2.68)	Reference
	IPTW	2.31 (1.43- 3.72)	Reference
Atelectasis requiring bronchoscopic intervention	Crude	2.69 (1.17–6.18)	Reference
	Adjusted^a^	2.77 (1.08–7.13)	Reference
	IPTW	2.64 (1.01–6.87)	Reference
Pneumonia	Crude	2.50 (1.75–3.58)	Reference
	Adjusted^a^	2.17 (1.49–3.17)	Reference
	IPTW	2.30 (1.10–4.82)	Reference
ALI/ARDS	Crude	2.72 (1.40–5.26)	Reference
	Adjusted^a^	2.22 (1.04–4.70)	Reference
	IPTW	2.70 (1.28–5.71)	Reference

Data are presented as a ratio (95% confidence interval).

PPCs, postoperative pulmonary complications; FEV1, forced expiratory volume in 1 s; %pred, %predicted; Quintile 1, the lowest quintile; Quintile 5, the top quintile; DLco, diffusing capacity of the lung for carbon monoxide, IPTW, inverse probability treatment weight.

^a^Adjusted for age, sex, body mass index, smoking status (never and ever), chronic pulmonary disease, cardiovascular disease, albumin, pathologic stage (I, II, and III), tumor location (cervical/upper thoracic, middle thoracic, and lower thoracic/esophagogastric junction), type of surgery (open thoracotomy, video-assisted thoracoscopic, and robotic surgery), lymph node dissection (two-field or less and three-field), operation time.

### The incidence of PPCs by DLco %pred

A trend of gradual increase in PPCs was observed as DLco %pred is decreased from Q5 to Q1 (*P* for trend < 0.01, Fig. 1b and Table 2). The increasing trend in the incidence of atelectasis requiring bronchoscopic toileting, pneumonia, and ALI/ARDS was observed according to FEV_1_%pred.

The RR of overall PPCs in Q2, Q3, Q4, and Q5 groups versus Q1 group of DLco %pred is summarized in Table 3. The RR of overall PPCs was highest in Q1 group of FEV_1_%pred followed by Q2, Q3, Q4, and Q5, and this trend remained significant after adjustment for covariables. Compared to high DLco group, the RR of overall PPCs and individual components of PPCs (atelectasis requiring bronchoscopic toileting, pneumonia, and ALI/ARDS) significantly higher in low DLco group. The results using the IPTW were similar to the multivariable-adjusted model.

### Joint effect of FEV_1_%pred DLco %pred for the occurrence of overall PPCs

The adjusted RR of overall PPCs was highest in patients with low DLco %pred and low FEV_1_%pred followed by low DLco %pred and high FEV_1_%pred, high DLco %pred and low FEV_1_%pred, and high DLco %pred and high FEV_1_%pred (Reference, Table 5). The results using the IPTW were similar to the multivariable-adjusted model.

**Table 5:** Joint effect of FEV_1_%pred and DLco %pred for the relative risk for overall PPCs.

	Crude	Adjusted^a^	IPTW
High DLco %pred & high FEV_1_%pred	Reference	Reference	Reference
High DLco %pred & low FEV_1_%pred	1.66 (1.02–2.59)	1.46 (0.96–2.20)	1.48 (0.95–2.33)
Low DLco %pred & high FEV_1_%pred	2.40 (1.58–3.56)	2.06 (1.42–2.99)	2.16 (1.51–3.10)
Low DLco %pred & low FEV_1_%pred	2.77 (1.74–4.25)	2.30 (1.53–3.44)	2.29 (1.54–3.40)

Data are presented as a ratio (95% confidence interval).

Cutoff values of low DLco %pred and low FEV_1_%pred are 72 and 78 respectively.

PPCs, postoperative pulmonary complications; FEV1, forced expiratory volume in 1 s; %pred, %predicted; Quintile 1, the lowest quintile; Quintile 5, the top quintile; DLco, diffusing capacity of the lung for carbon monoxide, IPTW, inverse probability treatment weight.

^a^Adjusted for age, sex, body mass index, smoking status (never and ever), chronic pulmonary disease, cardiovascular disease, albumin, pathologic stage (I, II, and III), tumor location (cervical/upper thoracic, middle thoracic, and lower thoracic/esophagogastric junction), type of surgery (open thoracotomy, video-assisted thoracoscopic, and robotic surgery), lymph node dissection (two-field or less and three-field), operation time.

## Discussion

In this retrospective cohort study in patients with esophageal cancer who underwent esophageal resection, we observed significant association between low levels of preoperative lung functions (DLco and FEV_1_) and the occurrence of PPCs: low FEV_1_ group had an approximately 1.5-fold increased risk of PPCs than the high FEV_1_ group and the risk of PPCs was approximately 2.0-fold higher in the low DLco group compared to the high DLco group. Importantly, when both lung function parameters were considered together, patients with both low DLco and low FEV_1_ showed 2.3-fold increased risk of developing PPCs compared to patients with both high DLco and high FEV_1_.

Our study expanded previous findings examining the predictive ability of preoperative lung function testing for PPCs in patients with esophageal cancer. Pulmonary function testing is commonly performed before not only for lung resection surgeries but also for extra-pulmonary surgeries to assess the risk of morbidity and mortality related to the surgery. Previous studies have shown that reduced lung function is an important contributor in predicting the occurrence of PPCs^8–12^. However, in the case of esophageal cancer, despite the higher risk of PPCs occurrence than in other surgeries^1–7^, only few studies have examined the association between preoperative lung function and PPCs after esophagectomy. For example, one previous study revealed that low FEV_1_ was associated with delayed weaning of mechanical ventilation: but this study was limited by an analysis of a small number of patients (n = 60) performed by a single surgeon, and this study did not evaluate PPCs other than delayed weaning of mechanical ventilation^5^. In 2018, Dutch group reported low DLco as an independent predictor of the major PPCs (Clavien-Dindo classification IIIb or higher: intervention requiring general anesthesia, life-threatening complications requiring intensive care, organ dysfunction, or death) after esophagectomy for esophageal cancer^17^. They suggested 85% as an ideal cutoff for DLco %pred. They also found preoperative FEV_1_%pred was significantly lower in patients presenting major PPCs (*P* = 0.011), but the significance did not remain in multivariable-adjusted model. Another study from the United States in patients with esophageal cancer treated with surgical resection after chemoradiation similarly showed a close relationship between PPCs and pre-treatment DLco, while pre-treatment FEV_1_ was related to the development of gastrointestinal complications^18^. Therefore, in agreement with and expanding upon previous findings, our results concerning the potential role of the preoperative values of DLco and FEV1 in the development of PPCs warrant further studies on constructing a predictive model for preventing PPCs.

One of notable approach in our study might be a joint effect analysis for PPCs. This approach incorporated a previous study that showed FEV_1_ and DLco were independently associated with PPCs after esophagectomy^16^. Our study has much larger numbers of study participants (n = 810 versus n = 516), and that study multiply imputed data because of large volume of missing data, which is not recommended method for handling missing values currently^19^. In addition, our study found that DLco plays a slightly more significant role than FEV_1_ in predicting PPCs after esophagectomy. While both FEV_1_ and DLco exhibited associations with post-esophagectomy PPCs in patients with esophageal cancer during individual analyses, statistical significance was achieved solely for DLco across all components of PPCs (atelectasis requiring bronchoscopic toileting, pneumonia, and ALI/ARDS). Moreover, in a fully adjusted model, DLco showed a larger effect size compared to FEV_1_. Several explanations could exist. First, DLco may show better performance over FEV_1_ by its ability to reflect general conditions of body as well as lung function itself. DLco can be influenced by body mass index, anemia, and nutritional conditions, whereas FEV_1_ is mainly influenced by the mechanics of the chest system^20–22^. Indeed, preoperative nutrition status, albumin level, as well as hand grip strength were closely associated with the development of post-esophagectomy PPCs^23–25^. Second, in terms of lung physiology, DLco could assess physiologic function of the lung more comprehensively than testing airflow (FEV_1_). For example, a reduced DLco could be related to obstructive lung diseases, as restrictive lung diseases, pulmonary vascular disorders, and other systemic diseases^26^. However, regarding this, since not much has been revealed, further studies are necessary. Our study findings may indicate that the strategies to prevent PPCs should consider preoperative measurement of DLco in patients with esophageal cancer who are planned to undergo esophagectomy.

Our study has several limitations. First, this study was performed in a single study with a retrospective design. Temporal causality might not be guaranteed. Second, we used the lowest quintile as a cutoff of FEV_1_ and DLco, and the cutoff values of FEV_1_%pred and DLco %pred were different. However, it was found that a sensitivity analysis using 80%pred as a cutoff of FEV_1_ and DLco showed similar results (Supplementary Table 1). Third, the patients in our study had relatively persevered pulmonary function. The mean values of FVC %pred, FEV_1_%pred, and DLco %pred were all > 80. Thus, our results might not be generalizable across all patients with esophageal cancer who underwent esophagectomy, and this warrants further study especially in patients with low lung function. Finally, we used smoking status as a binary variable. However, it should be noted that other confounders, such as pack-years, could affect the observed findings, which were not collected in our study.

In conclusion, reduced preoperative lung function, FEV_1_ and DLco, was significantly associated with an increased probability of PPCs after esophagectomy in patients with esophageal cancer. Decreased value of preoperative DLco seems to play a slightly more negative role for the development of PPCs than FEV_1_. In addition, there was more intensified association with PPCs when FEV_1_ and DLco were decreased together. Our study suggests that preoperative lung function could be useful for the stratification of patients at risk for PPCs who underwent esophagectomy for esophageal cancer.

## Methods

### Patients

**Figure 2:**
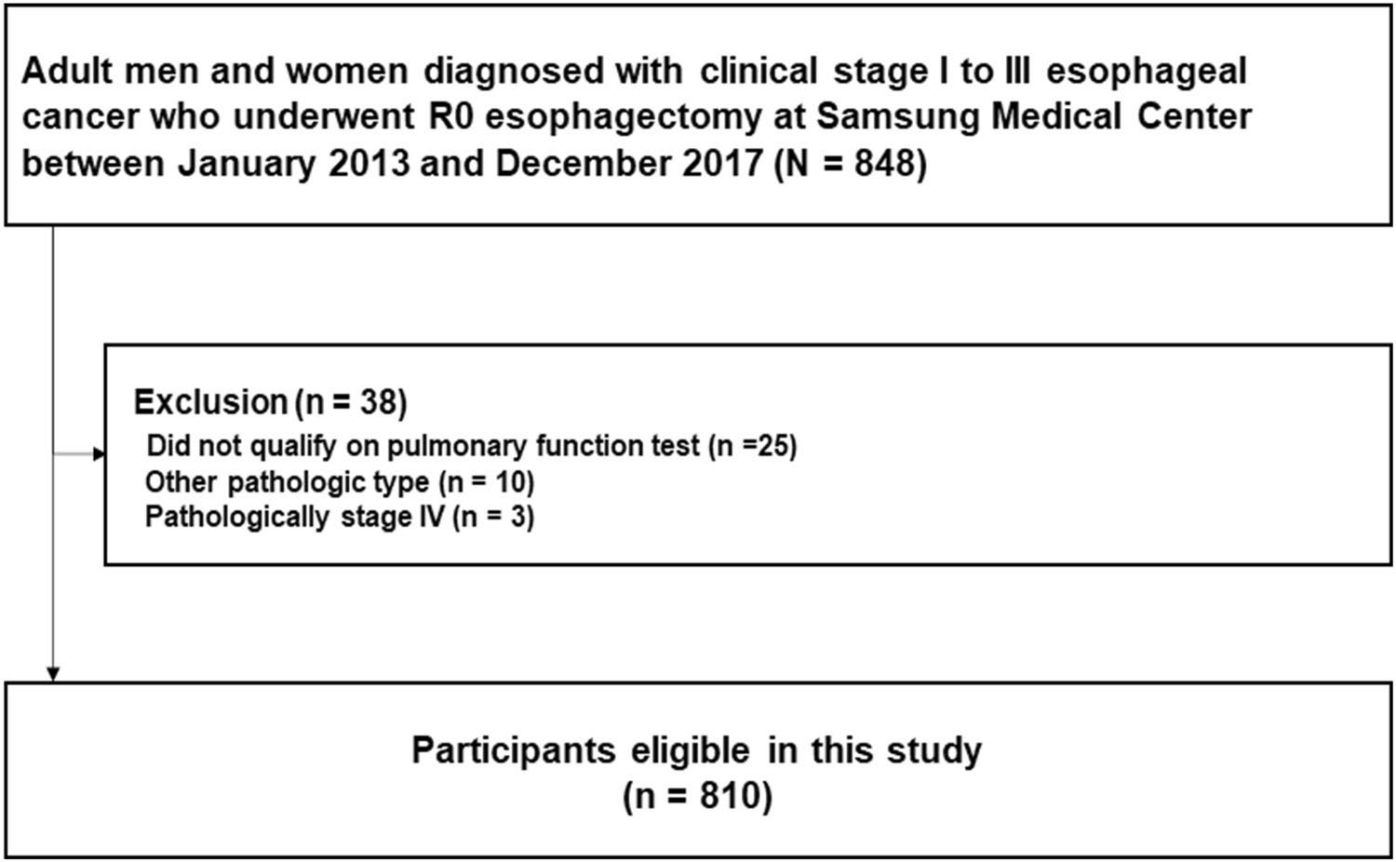
Flow chart of patients with esophageal cancer.

This study enrolled 848 patients with clinical stage I–III esophageal cancer who underwent curative R0 esophagectomy at Samsung Medical Center between January 2013 and December 2017. Patients who received neoadjuvant treatment were not included in this study. After excluding 25 patients who did not have preoperative lung function measurements, 10 patients with pathologic types other than squamous cell carcinoma ad adenocarcinoma, and 3 patients who were diagnosed with pathologically stage IV after esophagectomy, a total of 810 patients were analyzed (Fig. 2).

Institutional Review Board of Samsung Medical Center (IRB no. 2020-06-056) approved the study protocol and waived the informed consent from the participants since the nature of this study was retrospective and patient data were anonymized. This study was conducted in accordance with the Declaration of Helsinki. All procedures were performed in accordance with the relevant guidelines and regulations.

### Lung function measurements

Spirometry and DLco measurements were performed by using Vmax 22 (SensorMedics, CA, USA) according to the American Thoracic Society/European Respiratory Society criteria^27,28^. Absolute values of FEV_1_, forced vital capacity (FVC), and DLco were obtained, and the percentage of predicted values (% pred) for FEV_1_, FVC, and DLco was calculated by using a reference equation obtained on analysis of a representative South Korean sample^29,30^.

Since the optimal cutoff values of FEV_1_%pred and DLco %pred for PPCs after esophagectomy are not established, patients were classified into high and low pulmonary function groups based on the quintiles of pulmonary measurements. The high FEV_1_ group was defined as those with quintiles 2–5 (Q2–5) of FEV_1_%pred and the low FEV_1_ group as those with quintile 1 (Q1) of FEV_1_%pred. Similarly, high DLco group was defined as those with Q2–5 of DLco %pred and low DLco group as those with Q1 of DLco %pred.

### Other variables

Baseline demographics and behavioral information, including patient age, sex, body mass index, smoking status, comorbidities, and laboratory findings were collected through retrospective review. Information, including postoperative pathological stage, histological types, and surgical methods were also collected.

### PPCs

PPCs were defined as the occurrence of one or more of the followings after esophagectomy: (1) atelectasis requiring bronchoscopic toileting; (2) pneumonia (at least three among leukocytosis, pulmonary infiltrate or consolidation, fever [> 38 °C], culture-positive, or use of antibiotics); or (3) ALI/ARDS (PaO_2_/FiO_2_ < 300 and bilateral infiltrate seen on chest radiograph with no evidence of congestive heart failure or volume overload). All PPCs in this study were assessed by using the Clavien-Dindo classification^31^.

### Statistical analyses

Categorical variables were described as frequency and percentage, and continuous variables were described as median and interquartile range or mean and standard deviation. Categorical variables were compared using Pearson’s chi-squared test or Fisher’s test, as appropriate. Continuous variables were compared with the t-test or Mann–Whitney U test depending on the normality of the data.

We used a modified multivariable-adjusted Poisson regressive model to estimate the RR and confidence interval by using the robust error variances^32^. We adjusted for age, sex, smoking history (never and ever smoker), body mass index (kg/m^2^), the presence of pulmonary comorbidities (yes and no), the presence of cardiovascular comorbidities (yes and no), albumin (g/dL), pathologic stage (I, II, and III), tumor location (cervical/upper esophagus, mid esophagus, and low esophagus/esophagogastric junction), type of surgery (open thoracoscopic surgery, video-assisted thoracoscopic surgery, and robotic surgery), and extent of lymph node dissection (two or fewer locations and three locations), and surgical time (hours).

Subgroup analyses were performed to identify the association between preoperative lung function and specific types of PPCs; atelectasis requiring bronchoscopic toileting, pneumonia, and ALI/ARDS. Sensitivity analyses were conducted by 80%pred, a well-known practical cutoff value of FEV_1_ and DLco.

In addition, to investigate whether there is a joint effect with FEV_1_ and DLco on the relationship with PPCs, we further classified patients into four groups as follows: high FEV_1_/high DLco group, low FEV_1_/high DLco group, high FEV_1_/low DLco, and low FEV_1_/low DLco.

Besides of multivariable-adjusted Poisson model, an additional IPTW model was used to adjust for any potential group imbalances. To compute IPTW for multiple groups, a multinomial logit model was used to generate propensity score, and weights were assigned as the inverse of the probability of the groups (1/probability [treatment 0], 1/probability [treatment 1], 1/probability [treatment 2], etc.).

All tests were two-sided and a *P* < 0.05 was considered to be statistically significant. All analyses were performed using STATA version 15 (StataCorp, LP, USA).

### Ethical approval

Institutional Review Board of Samsung Medical Center (IRB no. 2020-06-056) approved the study protocol and waived the informed consent from the participants since the nature of this study was retrospective and patient data were anonymized. This study was conducted in accordance with the Declaration of Helsinki. All procedures were performed in accordance with the relevant guidelines and regulations.

### Supplementary Information

Supplementary Table 1.

## Data Availability

The datasets used and analyzed in the current study are available from the corresponding author upon reasonable request.
