# Author Correction: Preoperative DLco and FEV_1_ are correlated with postoperative pulmonary complications in patients after esophagectomy

**DOI:** 10.1038/s41598-024-58398-9

**Published:** 2024-04-02

**Authors:** Taeyun Kim, Yeong Jeong Jeon, Hyun Lee, Tae Ho Kim, Seong Yong Park, Danbee Kang, Yun Soo Hong, Genehee Lee, Junghee Lee, Sumin Shin, Jong Ho Cho, Yong Soo Choi, Jhingook Kim, Juhee Cho, Jae Ill Zo, Young Mog Shim, Hong Kwan Kim, Hye Yun Park

**Affiliations:** 1 Division of Pulmonary and Critical Care Medicine, Department of Medicine, Kosin University Gospel Hospital, Kosin University College of Medicine, Busan, South Korea; 2 Department of Thoracic and Cardiovascular Surgery, Samsung Medical Center, Sungkyunkwan University School of Medicine, 81 Irwon‑ro, Gangnam‑gu, Seoul, 06351 Republic of Korea; 3 Department of Internal Medicine, Hanyang University College of Medicine, Seoul, South Korea; 4 Department of Thoracic and Cardiovascular Surgery, Chung-Ang University Hospital, Seoul, South Korea; 5 Center for Clinical Epidemiology, Samsung Medical Center, Sungkyunkwan University School of Medicine, Seoul, South Korea; 6 Department of Epidemiology and Medicine, and Welch Center for Prevention, Epidemiology, and Clinical Research, Johns Hopkins Bloomberg School of Public Health, Baltimore, MD USA; 7 Patient-Centered Outcomes Research Institute, Samsung Medical Center, Seoul, South Korea; 8 Department of Clinical Research Design and Evaluation, SAIHST, Sungkyunkwan University, Seoul, South Korea; 9 Division of Pulmonary and Critical Care Medicine, Department of Internal Medicine, School of Medicine, Samsung Medical Center, Sungkyunkwan University, 81 Irwon‑ro, Gangnam‑gu, Seoul, 06351 Republic of Korea

Correction to: *Scientific Reports* 10.1038/s41598-024-56593-2, published online 13 March 2024

The Funding section in the original version of this Article was omitted. The Funding section now reads:

“This research was supported by a grant of the Korean Cancer Survivors Healthcare R&D Project through the National Cancer Center, funded by the Ministry of Health & Welfare, Republic of Korea (HA23C0515).”

The original Article has been corrected.

