# Supplementary material for: Preoperative DLco and FEV1 are correlated with postoperative pulmonary complications in patients after esophagectomy
Source: Sci Rep. 2024 Mar 13;14:6117. doi: 10.1038/s41598-024-56593-2 (PMC10937667; doi:10.1038/s41598-024-56593-2)
Supplement: Supplementary file 1 — Supplementary Table 1. [file 41598_2024_56593_MOESM1_ESM.docx]

**Supplemental Table 1.** The relative risk for PPCs comparing the low lung function group and the high lung function group using a different cutoff (80%pred of pulmonary measurements) in patients with esophageal cancer who underwent esophagectomy

|  | Model | FEV_1_ %pred | |
| --- | --- | --- | --- |
|  |  | <80  (n = 178) | ≥80  (n = 632) |
| Overall PPCs | Crude | 1.68 (1.26–2.24) | Reference |
|  | Adjusted* | 1.43 (1.06–1.93) | Reference |
| Atelectasis requiring bronchoscopic intervention | Crude | 1.33 (0.53–3.35) | Reference |
|  | Adjusted* | 1.41 (0.51–3.92) | Reference |
| Pneumonia | Crude | 1.94 (1.34–2.81) | Reference |
|  | Adjusted* | 1.68 (1.15–2.46) | Reference |
| ALI/ARDS | Crude | 1.48 (0.72–3.04) | Reference |
|  | Adjusted* | 1.30 (0.60–2.79) | Reference |
|  | Model | DLco % pred | |
|  |  | < 80  (n = 275) | ≥ 80  (n = 535) |
| Overall PPCs | Crude | 2.23 (1.70–2.94) | Reference |
|  | Adjusted* | 1.98 (1.48–2.67) | Reference |
| Atelectasis requiring bronchoscopic intervention | Crude | 2.33 (1.02–5.34) | Reference |
|  | Adjusted* | 2.17 (0.89–5.30) | Reference |
| Pneumonia | Crude | 2.67 (1.85–3.85) | Reference |
|  | Adjusted* | 2.38 (1.64–3.45) | Reference |
| ALI/ARDS | Crude | 4.67 (2.26–9.63) | Reference |
|  | Adjusted* | 4.20 (1.88–9.38) | Reference |

Data are presented as a ratio (95% confidence interval).

*Adjusted for age, sex, body mass index, smoking status (never and ever), chronic pulmonary disease, cardiovascular disease, albumin, pathologic stage (I, II, and III), tumor location (cervical/upper thoracic, middle thoracic, and lower thoracic/esophagogastric junction), type of surgery (open thoracotomy, video-assisted thoracoscopic, and robotic surgery), lymph node dissection (two-field or less and three-field), operation time

PPCs: postoperative pulmonary complications; FEV_1_: forced expiratory volume in 1 second; %pred: %predicted; Quintile 1: the lowest quintile; Quintile 5: the top quintile; ALI: acute lung injury; ARDS: acute respiratory distress syndrome; DLco: diffusing capacity of the lung for carbon monoxide.
